# Autoimmune encephalopathy associated with thyroid autoantibodies as the cause of reversible cognitive impairment

**DOI:** 10.3402/jchimp.v2i1.11453

**Published:** 2012-04-30

**Authors:** Natallia Maroz, Nechama Bernhardt, Robert Dobbin Chow

**Affiliations:** 1 Department of Medicine, Good Samaritan Hospital of Maryland, Baltimore, MD, USA; 2 Division of Nephrology, Hypertension and Renal Transplantation University of Florida, Gainesville, FL, USA; 3 Taylor Medical Group, Baltimore, Maryland, MD, USA

**Keywords:** hashimoto, reversible cognitive impairment, autoimmune encephalopathy, thyroid-peroxidase antibody

## Abstract

We herewith describe a patient with acute confusion, expressive aphasia and generalized seizures. A through workup excluded most causes of encephalopathy. He was, however, found to have TSH=18.6 MIU/ml, T3reverse=0.44nmol/L, T4=0.8ng/dl and Anti-Thyroid-Peroxidase AB titer >1000 IU/ml. Based on the above findings the patient was diagnosed with Hashimoto's encephalopathy and his mental status showed dramatic improvement (MMS 30/30) with high dose prednisone.

Hashimoto's encephalopathy is rare disorder of presumed autoimmune origin characterized by cognitive decline, seizures, neuro-psychiatric symptoms, high titers of Anti-Thyroid-Peroxidase AB, and a positive response to steroids.

We present a case of autoimmune encephalopathy associated with thyroid autoantibodies or so-called Hashimoto's Encephalopathy (HE). This is an uncommon and frequently underrecognized autoimmune disorder. Historically linked to autoimmune thyroiditis, the exact mechanism of disease is not known. High titers of thyroid autoantibodies are found at the time of diagnosis. However it is unclear whether antithyroid antibodies represent an immune epiphenomenon in a subset of patients with encephalopathic processes or are they associated with the underlying pathogenic mechanisms of the disorder (1). Though rare, it should be considered in the differential diagnosis of progressive dementia, particularly in a middle age population. Misdiagnosis of a potentially reversible condition as a progressive neurodegenerative disorder on the basis of the presumption of irreversibility can have devastating consequences for the patient and their family (2).

## Case

### Initial presentation and history

A 49-year-old African-American male with a past history of dyslipidemia and hypertension was admitted to the hospital on three separate occasions over a three-month period.

He was initially admitted with acute onset of cramping abdominal pain and diarrhea, and was treated for presumed viral gastroenteritis. His hospital course was complicated by delirium, development of sacral pressure ulcers due to prolonged sitting on a commode, and pre-renal acute renal failure. His renal function improved with hydration, but he required surgical debridement of his sacral decubiti. His mental status returned to normal in two days without specific intervention.

One month later, the patient was readmitted for acute confusion and difficulties in word finding. However, one day after the admission, the patient was examined by the consulting neurologist and was found to have a normal neurological exam without focal findings, including speech that was without aphasia or dysarthria. An evaluation for possible ischemic, infectious and metabolic etiologies revealed no abnormalities. The patient's symptoms resolved spontaneously over the ensuing three days and he was discharged with only mild residual cognitive deficits, which then resolved over the course of a week.

Two months later, he was admitted for a third time with an acute onset of confusion and change in speech.

### Physical examination

The patient was obese, with a Body Mass Index of 48.4. The examination was otherwise normal, aside from the neurological examination, which revealed normal cranial nerve and motor function, but a nonfluent aphasia was present. The patient was able to repeat short sentences and follow simple commands, but had difficulty with complex commands. He was able to answer some questions with simple phrases but most often answered with ‘ah ah ah’. Examination of the thyroid gland was suboptimal due his body habitus, but a goiter was not detected.

During this admission, he again experienced pre-renal acute renal failure, which was attributed to lack of oral intake and which improved with intravenous fluid administration. Though he had no prior history of seizure disorder, he had several witnessed generalized tonic-clonic seizures in the emergency department. He was moved to the Intensive Care Unit for monitoring, and was started on anti-epileptic medication.

### Laboratory and imaging studies

Laboratory data and complimentary studies are summarized in Tables 1 and 2 respectively. Potential causes of his cognitive impairment (e.g., metabolic, toxic, nutritional, neoplastic) were explored and ruled out.

**Table 1:** Laboratory studies

Variable	Result	Reference range,adults*
Hemoglobin (g/dl)	14.2	13.0–18.0
Hematocrit (%)	43.4	42.0–52.0
White cells (K/UL)	5.45	4.0–10.8
Platelets (k/UL)	261	150–400
Sodium (mmol/L)	142	137–145
Potassium (mmol/L)	4.0	3.6–5.0
Chloride (mmol/L)	106	98–107
Bicarbonate (mmol/L)	22	22–30
Blood urea nitrogen (mg/dl)	37	6.0–30.0
Creainine (mg/dl)	3.1	0.8–1.5
Glucose (mg/dl)	82	74–106
Calcium(mg/dl)	8.7	8.4–10.2
Phosphorus(mg/dl)	3.8	2.5–4.5
Magnesium(mg/dl)	2.2	1.6–2.3
Bilirubin total(mg/dl)	0.4	0.0–0.4
AST (U/L)	21	0–37
ALT (U/L)	32	0–41
Lactic acid (mmol/l)	1.9	0.7–2.1
ANA antibodies	Negative	Negative
Rheumatoid Factor	Negative	Negative
C-ANCA	Negative	Negative
p-ANCA	Negative	Negative
C3 (mg/dl)	71	90–180
C4 (mg/dl)	21	15–46
TSH (mlU/ml)	18.6	0.26–4.2
T4 free (ng/dl)	0.8	0.93–1.7
T3 reverse (pg/ml)	44	90–350
Thyroid-Peroxidase Ab(IU/ml)	>1000	Negative
Acetylcholine receptor binding Ab	Negative	Negative
Porphobilinogen (nmol/L/sec)	3	<6
Hepatitis B core Ab IgM	Negative	Negative
Hepatitis B surface Ag	Negative	Negative
Hepatitis C Ab	Negative	Negative
Hepatitis A Ab IgM	Negative	Negative
HIV PCR	Negative	Negative
Serum paraneoplastic panel:		
N-type CaCh ab (nmol/L)	0.00	0.00–0.03
GAD65 (nmol/L)	0.00	0.00–0.02
VGKC Ab (nmol/L)	0.00	0.00–0.02
Ceruloplasmin (mg/dl)	19	20–50
Vitamin B12 level (pg/ml)	789	243–984
Blood culture	No growth	No growth
Arterial blood gas:		
pH	7.35	7.35–7.45
PCO2 (mm Hg)	44	35.0–45.0
PO2 (mm Hg)	98	80.0–100.0
Bicarbonate (meq/l)	23	22.0–26.0
SatO2 (%)	100	90.0–100.0
FiO2 (%)	21	21–100
Troponin I (ng/ml)	0.00	0.00–0.12
CK-MB (ng/ml)	0.88	0.00–2.37
CPK (U/L)	23	55–170
Cerebrospinal fluid:		
WBC (cells/mm^3^) tube 1	8	0–10
RBC (cells/mm^3^) tube 1	152	0–3
RBC (cells/mm^3^) tube 4	2	0–3
Total protein (mg/dl) tube 1	84	15–45
Oligoclonal bands	Absent	Absent
Gram stain	Negative	Negative
Bacterial antigens	Negative	Negative
Herpes Simplex Viral titer	Negative	Negative
Cytomegalovirus titer	Negative	Negative
Lyme Ab	Negative	Negative
West Nile Ab	Negative	Negative

* Reference values are affected by many variables, including the patient population and the laboratory methods used. The ranges used at Good Samaritan Hospital are for adults who are not pregnant and do not have medical conditions that could affect the results. They may therefore not be appropriate for all patients.

Ab-antibodies; CaCh=voltage-gated calcium channel; VGKC= voltage-gated potassium channel; GAD65=glutamic acid decarboxylase-65

**Table 2:** Complimentary investigations

Investigations	Result
CAT scan of the head without contrast	No pathological findings reported
CAT scan of the chest without contrast	No pathological findings reported
CAT scan of the abdominal and pelvis without contrast	No pathological findings reported
Electroencephalogram	Mild symmetrical slowing in the theta range at 5–7 hertz, suggestive of diffuse encephalopathy
Holter monitoring for 24 hours	No arrhythmias detected
Electrocardiogram	Normal sinus rhythm
Magnetic Resonance Imaging of the brain	Not done due to morbid obesity
Ultrasound/Doppler of carotid arteries	No stenosis identified

He was found, however, to have high Thyroid-Peroxidase (TPO) antibodies concentration >1000 IU/ml (reference range: undetectable)

### Diagnosis and management

Based on the clinical picture of fluctuating cognitive status with subacute onset, seizures and high titer of TPO antibodies, the diagnosis of autoimmune encephalopathy was made. High dose prednisone was initiated, which was followed by significant improvement in his cognitive function (Mini-Mental State Examination (MMSE) changed from 23/30 to 30/30) over a course of two weeks. It is important to mention that thyroid function replacement therapy was not instituted.

### Outcome and follow up

In the outpatient settings, the dose of prednisone was gradually reduced from 40 mg to 5 mg a day over the course of six months. His mental status and social functioning returned to baseline, and he was able to resume his usual work duties as a security officer. One year after his last hospitalization, he had no further relapses while on maintenance dose of prednisone 5 mg per day.

## Discussion

Physicians working in hospitals frequently encounter patients with cognitive impairment. The first step in evaluation of cognitive function is an assessment of mental status. As such, familiarity with various tools of mental status assessment (e.g., MMES) is essential for the house staff and physicians (3).

Searching for reversible causes of cognitive decline in the appropriate clinical settings, is the single most important diagnostic goal. It is worthwhile to perform more extensive work-up in cases of dementia. The cost of these investigations is small compared to the cost of the care for the disabled patient with potentially reversible condition (4). Moreover, unraveling and managing potentially reversible cognitive impairment is gratifying experiences for the physician and can make a significant difference for the lives of patients and their families.

The differential diagnosis for cognitive decline is broad. Thorough history and physical examination will narrow and focus the list of potential diagnoses. It is important to identify the duration of cognitive decline, nature of its progression, precipitating event (if any), comorbid conditions, and the extent of cognitive domains involved (i.e., memory, language, spatial skills, judgment and problem solving). Clues suggesting a reversible process include abrupt onset, disorientation, poor attention, altered level of consciousness, hallucinations and hypersomnolence. New onset of ataxia, clonus, tremor and hyperreflexia are frequently observed in reversible cases of cognitive impairment. Table 3 summarizes the list of conditions that should be considered in differential diagnosis.

**Table 3:** Differential diagnosis of new onset cognitive impairment in adult

Etiology	Diagnostic clues
**Nonreversible causes**	
Change of intracranial anatomy (e.g., trauma, tumor)	History. Confirmation by neuroimaging
Ischemic and non-ischemic stroke Subcortical vascular dementia	History. Confirmation by neuroimaging Gradual step-wise progression. Confirmation by neuropsychological testing
Incurable cerebral neoplasia, meningeal carcinomatosis	Frequently associated with systemic malignancy. Confirmation by neuroimaging, biopsy.
Alzheimer dementia	Gradual progression. Loss of memory, anomia, constructional apraxia, anosognosia, personality changes. Confirmation by neuropsychological testing.
Frontotemporal lobar dementia	Gradual progression. Nonfluent aphasia, deficit in abstraction, and executive function. Behavior and personality changes. Confirmation by neuropsychological testing.
Progressive supranuclear palsy	Gradual progression Abnormal extraocular movement, limitation of vertical gaze, gait imbalance, personality changes. Confirmation by neurological examination
Dementia with Lewy bodies	Gradual progression. Parkinsonism, visual hallucinations, constructional apraxia. Gait-balance disorder and delusions, sensitivity to traditional antipsychotics, and fluctuations in alertness. Confirmation by neuroimaging
Prion diseases	Presence of movement disorders, myoclonus. History of potential exposure to prion. Confirmation by characteristic electroencephalographic pattern of periodic sharp wave complexes and the pathologic examination of brain tissue
**Potentially reversible causes**	
Endocrine	Hyper or hypoglycemia, hypothyroidism, Addison disease Confirmed by measurement of hormone levels
Metabolic	Liver, renal, pulmonary and heart failure Confirmed by laboratory markers
Infectious:	
Systemic	-UTI, pneumonia, sepsis
Central Nervous System involvement	- Intracranial abscess, meningitis, encephalitis. CSF consistent with bacterial, fungal viral infection. (e.g. Syphilis, HIV infection, Lyme neuroborreliosis, Herpes, or, more rarely, Whipples disease) Confirmed by microbiological testing, imaging
Genetic disordes (eg. Wilson disease, Goucher disease, MELAS, Leigh syndrome, porphyria etc) Huntington's	Multiple organ involvement is frequent.Confirmation by genetic testing and biomarkers and neurological examination. Currently therapies available for several of the condition listed (with exception to Huntington's disease)
Inflammatory	Cerebral vasculitis. Confirmation by serological markers (e.g. ANA, pANCA, ESR), biopsy
Normal pressure hydrocephalus	Apraxia, gait disorder and urinary incontinence Confirmation by neuroimaging, CSF pressure and improvement in response to fluid withdrawal
Epileptic	Ictal and post-ictal state. Confirmed by EEG monitoring.
Therapeutic	Acute or chronic drug effect (e.g. lithium) History of intake
Nutritional	Wernicke encephalopathy, Beriberi, Pellagra Confirmed by laboratory investigations
Curable neoplastic conditions	Confirmed by neuroimaging, biopsy
Psychiatric	Acute or chronic psychosis, depression confirmed by psychiatric evaluation
Toxic	Exposure to toxic substance (e.g. lead, mercury), illegal substances
Subdural hematoma	Presentation varies depending from location. Frequent lethargy. Confirmation by neuroimaging
Paraneoplastic syndrome, limbic encephalitis	Associated with underlying malignancy, Confirmed by presence of anti-neural antibodies Not responsive to immunotherapy.
Autoimmune dementias characterized by specific syndromic presentation, specific serologic marker (e.g. thyroid autoantibody–associated HE), histopathologic findings	Fluctuating course is common. Frequent hypersomnolence, tremor, myoclonus, seizures, and psychiatric features. Presence of neural or antithyroid antibodies, inflammatory CSF. Neuroimaging atypical for a neurodegenerative dementia. Positive response to immunotherapy

Here we present a case of HE which is considered a rare but reversible case of cognitive impairment.

HE is defined as autoimmune dementia associated with thyroid autoantibodies and characterized by subacute or fluctuating cognitive impairment, high titers of TPO antibodies, and a positive response to immunotherapy (2). Other frequently observed features include tremor, myoclonus, seizures, headache, hypersomnolence and psychiatric symptoms (5). It was originally described by Lord Brian et al. in 1966 in a patient with encephalopathy, Hashimoto thyroiditis, and positive TPO antibodies (6). He linked these conditions and called the new syndrome HE. Since that time, more than 120 cases of immunotherapy-responsive encephalopathy associated with thyroid autoantibodies have been reported in the medical literature.

As seeing in most autoimmune diseases, HE is more prevalent in white females with a mean age of onset 45–55 years old. It has been reported in pediatric, adult and elderly populations throughout the world (7). Patients are frequently found to have coexisting autoimmune disorder.

The clinical presentation can be heterogeneous. HE most commonly presents as either a relapsing remitting encephalopathy or with stroke-like episodes. The remainder of the cases present with a wide spectrum of progressive psychiatric symptoms, including memory loss, delusions, agitation, visual hallucinations and social isolation (8). Seizures are present in 25–65% of cases and can be generalized (especially myoclonic) or focal.

The underlying pathophysiology of HE is not well delineated. It does not appear to be directly related to the dysfunction of the thyroid gland as the majority of the patients are clinically and biochemically euthyroid (5, 9). It is unclear whether antithyroid antibodies represent an immune epiphenomenon in a subset of patients with encephalopathic processes or they have direct association with pathogenic mechanisms of the disorder (2). Since these antibodies can also be detected in the healthy general population (1, 2) the presence of clinical features of autoimmune encephalopathy is crucial to make the correct diagnosis. Ferracci et al. reported presence of TPO antibodies and circulating immune complexes in the CSF of six patients with HE (10). In general, TPO antibody targets thyroid peroxidase, which is located inside the follicular cell and catalyzes the thyroglobulin iodination process in the production of the thyroid hormones. There is no established correlation between the titer of TPO and severity of autoimmune dementia, or its response to the therapy. The confusing nomenclature applied to autoimmune encephalopathies with cognitive impairment reflects the evolution in understanding of these disorders (2). Currently there is no universally-accepted classification available for the variety of these conditions. Several classification have been proposed based on a syndromic presentation (e.g., progressive encephalomyelopathy with rigidity and myoclonus), a specific serologic marker (e.g., thyroid autoantibody–associated HE, voltage-gated potassium channel [VGKC] complex antibody–associated), or histopathologic findings (e.g, nonvasculitic autoimmune meningoencephalitis). The potential for reversibility by immunotherapy unifies these disorders (2).

The differential diagnosis is usually broad and the evaluation requires excluding other causes of progressive encephalopathies (Table 3). The clinical picture, elevation of TPO antibodies, and positive response to corticosteroid therapy are essential for making the diagnosis of HE. Though the cortical and subcortical system may appear to be clinically involved in these cases, brain Magnetic Resonance Imaging (MRI) studies are usually unremarkable. MRI was not done in this case because of morbid obesity. Some authors have reported, however, that the MRI appearance of HE may sometimes simulate an ischemic stroke, atrophy, multiple tumors, granulomas, or even a degenerative process (11–13). They also reported evolutions of these lesions in response to immunotherapy (11–13). EEG findings are heterogeneous and can present with generalized slowing, frontal rhythmic slowing as well as focal left temporal slowing (14). Postmortem examinations are rare but perivascular lymphocytic infiltration throughout the brain and leptomeninges, diffuse gliosis of gray matter in the cortex, basal ganglia, thalami, hippocampus, and, to a lesser extent, the white matter have been described (15).

Treatment with immunotherapy is reported to be successful in as many as 64% of patients with autoimmune dementias, although relapses occur frequently after discontinuation of treatment. Non-responders to therapy were frequently found to have degenerative or prion disorder (2). Use of high doses of oral prednisone (50–150 mg/day), intravenous methylprednisolone (1 g/day for 3–7 days) (16), intravenous immune-globulin and plasmapheresis, all have been reported as therapeutic options for initial treatment. Long-term maintanace immunosuppression with prednisone and corticosteroid-sparing agents (i.e., azathioprine, mycophenolate mofetil, rituximab, methotrexate, and cyclophosphamide), have being used up to 108 months to attain long-term remission (2). Use of immunotherapy requires close follow up and prophylaxis measures for prevention of infectious complications. The risk of developing malignancies from long-term use of immunosuppressive agents in this population has not been investigated, but needs to be taken into consideration.

## Conclusion

Autoimmune encephalopathy with thyroid autoantibodies is a rare, life-threatening, and potentially treatable condition (9). It should be included in the differential diagnosis of patients with cognitive impairment. Prompt diagnosis and treatment of immunotherapy-responsive encephalopathy can dramatically change the course of the disease and improve the patient's prognosis.
